# Melaminium hydrogen malonate

**DOI:** 10.1107/S1600536812033016

**Published:** 2012-07-25

**Authors:** Barbara Froschauer, Matthias Weil

**Affiliations:** a Institute for Applied Synthetic Chemistry, Vienna University of Technology, Getreidemarkt 9/163, A-1060 Vienna, Austria; b Institute for Chemical Technologies and Analytics, Division of Structural Chemistry, Vienna University of Technology, Getreidemarkt 9/164-SC, A-1060 Vienna, Austria

## Abstract

The melaminium (2,4,6-triamino-1,3,5-triazin-1-ium) cation in the title compound, C_3_H_7_N_6_^+^·C_3_H_3_O_4_^−^, is essentially planar, with a r.m.s. deviation of the non-H atoms of 0.0085 Å. Extensive hydrogen bonding of the types N—H⋯N and N—H⋯O between cations and cations and between cations and hydrogen malonate (2-carb­oxy­ethano­ate) anions leads to the formation of supra­molecular layers parallel to (1-2-1). An intra­molecular O—H⋯O hydrogen bond in the single deprotonated malonate anion also occurs.

## Related literature

For the use of melaminium salts in polymer science, see: Weinstabl *et al.* (2001 ▶). For structural studies of melaminium salts of purely organic carb­oxy­lic acids, see: Choi *et al.* (2004 ▶); Janczak & Perpétuo (2001 ▶, 2002 ▶, 2003 ▶, 2004 ▶); Karle *et al.* (2003 ▶); Marchewka *et al.* (2003 ▶); Perpétuo & Janczak (2002 ▶, 2005 ▶); Perpétuo *et al.* (2005 ▶); Prior *et al.* (2009 ▶); Su *et al.* (2009 ▶); Udaya Lakshmi *et al.* (2006 ▶); Froschauer & Weil (2012 ▶); Zhang *et al.* (2004 ▶, 2005 ▶).
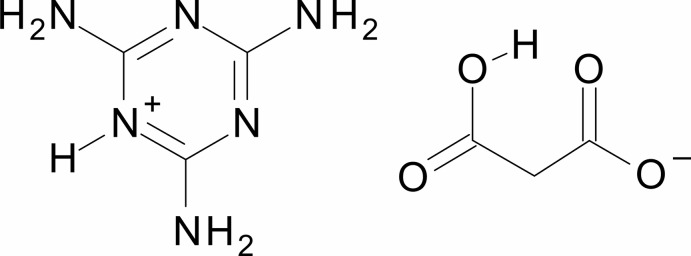


## Experimental

### 

#### Crystal data

C_3_H_7_N_6_^+^·C_3_H_3_O_4_^−^
*M* *_r_* = 230.20
Triclinic, 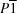

*a* = 5.1996 (15) Å
*b* = 7.499 (2) Å
*c* = 13.119 (4) Å
α = 100.206 (5)°
β = 98.014 (5)°
γ = 106.534 (5)°
*V* = 472.7 (2) Å^3^
*Z* = 2
Mo *K*α radiation
μ = 0.14 mm^−1^
*T* = 293 K
0.23 × 0.18 × 0.12 mm

#### Data collection

Siemens SMART CCD diffractometer
4807 measured reflections
2354 independent reflections
1190 reflections with *I* > 2σ(*I*)
*R*_int_ = 0.078

#### Refinement

*R*[*F* ^2^ > 2σ(*F* ^2^)] = 0.042
*wR*(*F* ^2^) = 0.105
*S* = 0.89
2354 reflections
150 parameters
H atoms treated by a mixture of independent and constrained refinement
Δρ_max_ = 0.23 e Å^−3^
Δρ_min_ = −0.23 e Å^−3^

### 

Data collection: *SMART* (Siemens, 1996 ▶); cell refinement: *SAINT* (Siemens, 1996 ▶); data reduction: *SAINT*; program(s) used to solve structure: *SHELXS97* (Sheldrick, 2008 ▶); program(s) used to refine structure: *SHELXL97* (Sheldrick, 2008 ▶); molecular graphics: *Mercury* (Macrae *et al.*, 2006 ▶) and *ATOMS* (Dowty, 2006 ▶); software used to prepare material for publication: *publCIF* (Westrip, 2010 ▶).

## Supplementary Material

Crystal structure: contains datablock(s) I, global. DOI: 10.1107/S1600536812033016/cv5323sup1.cif

Structure factors: contains datablock(s) I. DOI: 10.1107/S1600536812033016/cv5323Isup2.hkl

Supplementary material file. DOI: 10.1107/S1600536812033016/cv5323Isup3.cml

Additional supplementary materials:  crystallographic information; 3D view; checkCIF report

## Figures and Tables

**Table 1:** Hydrogen-bond geometry (Å, °)

*D*—H⋯*A*	*D*—H	H⋯*A*	*D*⋯*A*	*D*—H⋯*A*
N1—H1⋯O4^i^	0.86	1.82	2.6785 (19)	176
N4—H2⋯O2^ii^	0.86	2.17	2.8350 (19)	134
N4—H3⋯N2^ii^	0.86	2.14	2.994 (2)	171
N5—H4⋯O2	0.86	2.14	2.998 (2)	172
N5—H5⋯N3^iii^	0.86	2.23	3.091 (2)	178
N6—H6⋯O1^iii^	0.86	2.15	2.8592 (19)	140
N6—H7⋯O3^i^	0.86	2.02	2.880 (2)	173
O1—H10⋯O3	1.00 (2)	1.47 (2)	2.450 (2)	165 (2)

Symmetry codes: (i) 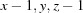; (ii) 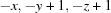; (iii) 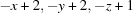.

**Fig. 1.**
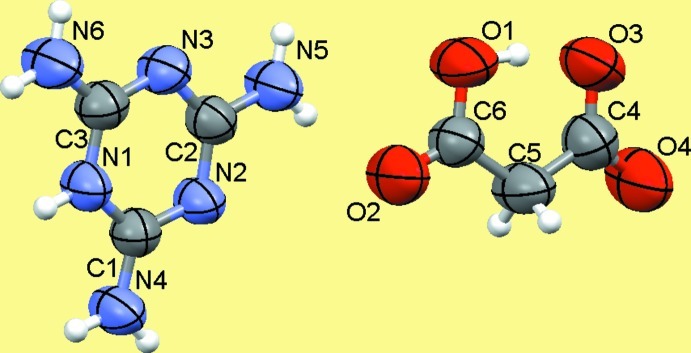
The molecular components of the title compound with displacement parameters drawn at the 90% probability level. H atoms are displayed as spheres with an arbirtary radius.

**Fig. 2.**
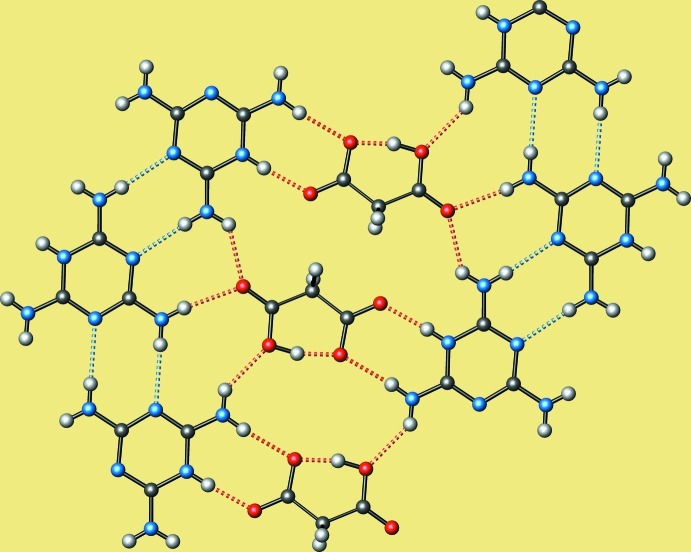
Supramolecular layer built up through hydrogen bonding interactions (dashed lines) between cations and cations and between cations and anions.

**Fig. 3.**
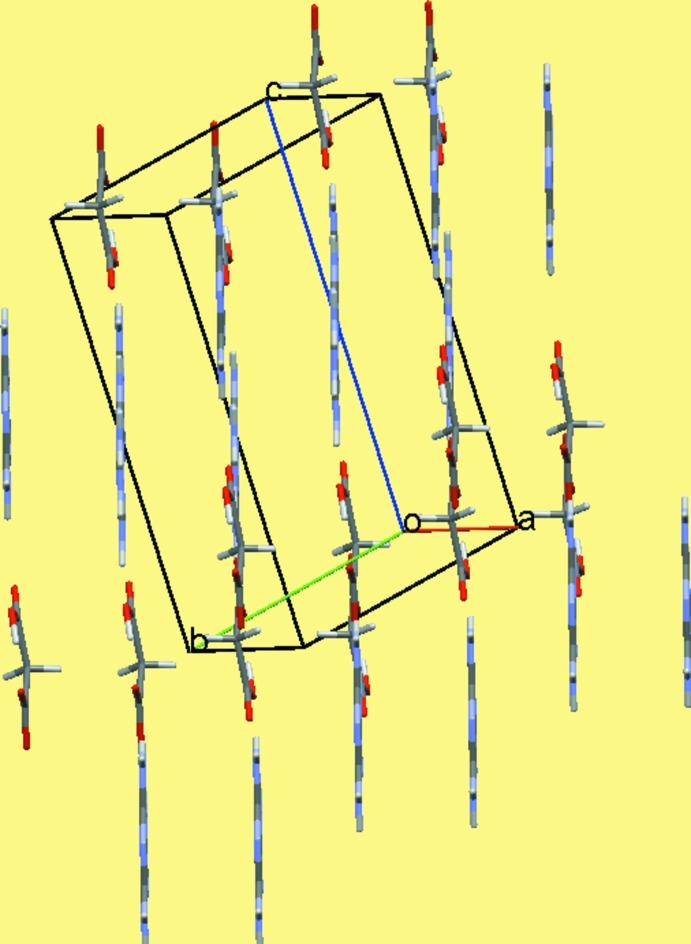
The assembly of supramolecular layers in the crystal parallel to (121).

## supplementary crystallographic information

### Comment

Melamine is a weak base with three different *pK_a_* values which decline with decreasing protonation status. The first (*pK_a_* = 5.10) is slightly above the *pK_a_* of acetic acid (4.75), the second and third (0.20 and -2.10, respectively) are significantly below the most organic carboxylic acids. Since the difference between the *pK_a_* values during an acid-base reaction corresponds to the free energy of reaction, stable products can only be expected for acids with a *pK_a_* value significantly below 5.10, whereas organic acids with acidities in the range of 5.10 or above can be expected to yield mixtures of unreacted melamine, free acid and melaminium salts. Depending on the acid valency and strengths, mono and disalts can be formed by simply heating the components or their respective solutions.

In the past, organic melamine salts were tested as potential melamine substitutes for melamine urea formaldehyde (MUF) resins (Weinstabl *et al.*, 2001). Up to now, the following melaminium salts of purely organic carboxylic acids, *viz* only those with C, H and N contents, have been crystallographically characterized: melaminium acetate acetic acid solvate monohydrate (Perpétuo & Janczak, 2002), melaminium 2,4,6-trihydroxybenzoate dihydrate (Prior *et al.*., 2009), melaminium benzoate dihydrate (Perpétuo & Janczak, 2005), melaminium formate (Perpétuo *et al.*, 2005), melaminium glutarate monohydrate (Janczak & Perpétuo, 2002), melaminium levulinate monohydrate (Choi *et al.*, 2004), melaminium maleate monohydrate (Janczak & Perpétuo, 2004), bis(melaminium) DL-malate tetrahydrate (Janczak & Perpétuo, 2003), melamin(1,3)ium dihydrogenmellitate dihydrate (Karle *et al.*., 2003), melaminium bis(hydrogen oxalate) (Zhang *et al.*, 2005), melaminium hydrogenphtalate (Janczak & Perpétuo, 2001), bis(melaminium) succinate succinic acid solvate dihydrate (Froschauer & Weil, 2012), melamin(1,3)ium tartrate monohydrate (Marchewka *et al.*, 2003), bis(melaminium) tartrate 2.5-hydrate (Udaya Lakshmi *et al.*, 2006), bis(melaminium) tartrate dihydrate (Su *et al.*, 2009), and bis(melaminium) terephtalate dihydrate (Zhang *et al.*, 2004).

The *pK_a_* values of 2.82 and 5.69 for the first and second deprotonation step of malonic acid led to a single deprotonated anion in the title compound, melaminium hydrogen malonate, C_3_H_7_N_6_^+.^C_3_H_3_O_4_^-^. The protonation of melamine takes place at one of the triazine N ring atoms (Fig. 1) as observed for all other single protonated melaminium salts listed above.

Both the melaminium cation and the hydrogenmalonate anions are essentially planar with r.m.s. deviations of 0.0085 Å (cation) and 0.061 Å (anion) for the non-H atoms. The angle between the two least-squares planes is 6.61 (8) °, making it possible to set up supramolecular layers held together by strong to medium hydrogen bonds of the type N—H···O and N—H···.N between cations and cations and cations and anions (Fig. 2; Table 1). The motif for the hydrogen-bonded assembly of two melaminium cations is observed in many other melamine or melaminium structures as reported previously by Prior *et al.* (2009). In the crystal, the supramolecular layers are arranged parallel to (121) (Fig. 3) with an interplanar distance of approximately 2.96 Å.

### Experimental

39.6 mmol melamine was dissolved under refluxing conditions in 150 ml distilled water. The stoichiometric quantity (1:1) of malonic acid was added within five minutes. The mixture was then refluxed for 30 minutes and then cooled to room temperature. The precipitate formed on cooling was separeted by filtration and washed with cold methanol. The crystalline product was then dried *in vacuo* at 303–313 K. Single crystal growth was accomplished by dissolution of 1 g of the crystalline product under refluxing conditions in an aqueous methanol solution (2:1 *v*/*v*) to get a saturated solution. Then the solution was slowly cooled down to room temperature. Suitable crystals were obtained by slow evaporation of the solvents during five days. The crystals were washed with methanol and dried *in vacuo* at room temperature giving analytical pure samples. CHN analysis (found/calc.): C (31.19/31.30), H (4.01/4.37), N (36.26/36.50). NMR: (solution, DMSO) chemical shift [p.p.m.]: ^1^H 11.04 (s, 2H), 6.97 (s, 6H), 3.01 (s, 2H); ^13^C 170.45, 163.38, 40.95.

### Refinement

The proton at the triazine ring of the melaminium cation was clearly discernible from a difference Fourier map (like all other H atoms). For refinement, the H atoms attached to C or N atoms were set in calculated positions and treated as riding on their parent atoms with C—H = 0.97 Å and N—H = 0.86 Å and with *U*_iso_(H) = 1.2*U*_eq_(C,*N*). The remaining proton of the carboxyl group was refined freely.

### Crystal data

**Table:**

C_3_H_7_N_6_^+^·C_3_H_3_O_4_^−^	*Z* = 2
*M**_r_* = 230.20	*F*(000) = 240
Triclinic, *P*1	*D*_x_ = 1.617 Mg m^−^^3^
Hall symbol: -P 1	Mo *K*α radiation, λ = 0.71073 Å
*a* = 5.1996 (15) Å	Cell parameters from 950 reflections
*b* = 7.499 (2) Å	θ = 2.9–25.6°
*c* = 13.119 (4) Å	µ = 0.14 mm^−^^1^
α = 100.206 (5)°	*T* = 293 K
β = 98.014 (5)°	Irregular, colourless
γ = 106.534 (5)°	0.23 × 0.18 × 0.12 mm
*V* = 472.7 (2) Å^3^	

### Data collection

**Table:**

Siemens SMART CCD diffractometer	1190 reflections with *I* > 2σ(*I*)
Radiation source: fine-focus sealed tube	*R*_int_ = 0.078
Graphite monochromator	θ_max_ = 28.5°, θ_min_ = 2.9°
ω scans	*h* = −6→6
4807 measured reflections	*k* = −10→10
2354 independent reflections	*l* = −17→17

### Refinement

**Table:**

Refinement on *F*^2^	Secondary atom site location: difference Fourier map
Least-squares matrix: full	Hydrogen site location: inferred from neighbouring sites
*R*[*F*^2^ > 2σ(*F*^2^)] = 0.042	H atoms treated by a mixture of independent and constrained refinement
*wR*(*F*^2^) = 0.105	*w* = 1/[σ^2^(*F*_o_^2^) + (0.038*P*)^2^] where *P* = (*F*_o_^2^ + 2*F*_c_^2^)/3
*S* = 0.89	(Δ/σ)_max_ < 0.001
2354 reflections	Δρ_max_ = 0.23 e Å^−^^3^
150 parameters	Δρ_min_ = −0.23 e Å^−^^3^
0 restraints	Extinction correction: *SHELXL97* (Sheldrick, 2008), Fc^*^=kFc[1+0.001xFc^2^λ^3^/sin(2θ)]^-1/4^
Primary atom site location: structure-invariant direct methods	Extinction coefficient: 0.058 (10)

### Special details

**Table:**

Geometry. All e.s.d.'s (except the e.s.d. in the dihedral angle between two l.s. planes) are estimated using the full covariance matrix. The cell e.s.d.'s are taken into account individually in the estimation of e.s.d.'s in distances, angles and torsion angles; correlations between e.s.d.'s in cell parameters are only used when they are defined by crystal symmetry. An approximate (isotropic) treatment of cell e.s.d.'s is used for estimating e.s.d.'s involving l.s. planes.
Refinement. Refinement of *F*^2^ against ALL reflections. The weighted *R*-factor *wR* and goodness of fit *S* are based on *F*^2^, conventional *R*-factors *R* are based on *F*, with *F* set to zero for negative *F*^2^. The threshold expression of *F*^2^ > σ(*F*^2^) is used only for calculating *R*-factors(gt) *etc*. and is not relevant to the choice of reflections for refinement. *R*-factors based on *F*^2^ are statistically about twice as large as those based on *F*, and *R*- factors based on ALL data will be even larger.

### Fractional atomic coordinates and isotropic or equivalent isotropic displacement parameters (Å^2^)

**Table:**

	*x*	*y*	*z*	*U*_iso_*/*U*_eq_	
H10	1.131 (4)	0.844 (3)	0.9236 (18)	0.068 (7)*	
O1	1.0512 (3)	0.8287 (2)	0.84785 (11)	0.0556 (5)	
N1	0.2299 (3)	0.7179 (2)	0.29981 (11)	0.0321 (4)	
H1	0.1159	0.6950	0.2412	0.038*	
N2	0.3254 (3)	0.6667 (2)	0.47008 (11)	0.0313 (4)	
O2	0.6330 (3)	0.6777 (2)	0.75923 (10)	0.0490 (4)	
N3	0.6677 (3)	0.8814 (2)	0.40391 (11)	0.0322 (4)	
N4	−0.0943 (3)	0.5082 (2)	0.36078 (11)	0.0385 (4)	
H3	−0.1474	0.4505	0.4089	0.046*	
H2	−0.2036	0.4858	0.3011	0.046*	
C1	0.1545 (3)	0.6307 (2)	0.37848 (13)	0.0294 (4)	
C3	0.4874 (3)	0.8417 (2)	0.31493 (14)	0.0311 (4)	
C5	0.7245 (4)	0.6701 (3)	0.94033 (14)	0.0429 (5)	
H8	0.6323	0.5333	0.9229	0.051*	
H9	0.5920	0.7311	0.9605	0.051*	
C2	0.5776 (3)	0.7909 (2)	0.47902 (13)	0.0288 (4)	
N6	0.5500 (3)	0.9219 (2)	0.23620 (11)	0.0452 (5)	
H6	0.7104	1.0015	0.2423	0.054*	
H7	0.4304	0.8946	0.1787	0.054*	
N5	0.7554 (3)	0.8292 (2)	0.56860 (11)	0.0387 (4)	
H4	0.7090	0.7756	0.6190	0.046*	
H5	0.9175	0.9078	0.5766	0.046*	
C6	0.7986 (3)	0.7256 (3)	0.84124 (14)	0.0337 (5)	
O4	0.8950 (3)	0.6463 (2)	1.11234 (10)	0.0585 (5)	
C4	0.9489 (4)	0.7151 (3)	1.03630 (15)	0.0394 (5)	
O3	1.1859 (2)	0.8250 (2)	1.03379 (10)	0.0528 (4)	

### Atomic displacement parameters (Å^2^)

**Table:**

	*U*^11^	*U*^22^	*U*^33^	*U*^12^	*U*^13^	*U*^23^
O1	0.0362 (8)	0.0771 (11)	0.0396 (9)	−0.0090 (7)	0.0065 (7)	0.0240 (8)
N1	0.0280 (8)	0.0396 (9)	0.0232 (8)	0.0021 (7)	0.0000 (6)	0.0118 (7)
N2	0.0264 (8)	0.0365 (9)	0.0258 (8)	0.0008 (7)	0.0023 (6)	0.0110 (7)
O2	0.0391 (8)	0.0689 (10)	0.0324 (8)	0.0037 (7)	0.0010 (6)	0.0218 (7)
N3	0.0280 (8)	0.0395 (9)	0.0275 (8)	0.0042 (6)	0.0043 (6)	0.0153 (7)
N4	0.0290 (8)	0.0480 (10)	0.0293 (9)	−0.0025 (7)	−0.0005 (7)	0.0152 (7)
C1	0.0274 (9)	0.0309 (10)	0.0273 (10)	0.0048 (8)	0.0050 (8)	0.0075 (8)
C3	0.0284 (10)	0.0324 (10)	0.0307 (11)	0.0055 (8)	0.0061 (8)	0.0098 (8)
C5	0.0295 (10)	0.0590 (13)	0.0356 (11)	0.0024 (9)	0.0034 (8)	0.0208 (10)
C2	0.0253 (9)	0.0306 (10)	0.0281 (10)	0.0065 (8)	0.0017 (8)	0.0077 (8)
N6	0.0338 (9)	0.0611 (11)	0.0309 (9)	−0.0052 (8)	0.0003 (7)	0.0232 (8)
N5	0.0307 (8)	0.0493 (10)	0.0286 (9)	−0.0019 (7)	−0.0002 (7)	0.0183 (7)
C6	0.0288 (10)	0.0397 (11)	0.0312 (11)	0.0064 (8)	0.0044 (9)	0.0132 (9)
O4	0.0481 (9)	0.0789 (11)	0.0345 (8)	−0.0047 (8)	−0.0037 (7)	0.0274 (8)
C4	0.0344 (11)	0.0459 (12)	0.0330 (11)	0.0065 (9)	0.0033 (8)	0.0100 (9)
O3	0.0327 (8)	0.0700 (10)	0.0404 (9)	−0.0041 (7)	−0.0012 (6)	0.0140 (7)

### Geometric parameters (Å, º)

**Table:**

O1—C6	1.303 (2)	C3—N6	1.318 (2)
O1—H10	0.99 (2)	C5—C6	1.501 (2)
N1—C3	1.359 (2)	C5—C4	1.509 (3)
N1—C1	1.361 (2)	C5—H8	0.9700
N1—H1	0.8600	C5—H9	0.9700
N2—C1	1.326 (2)	C2—N5	1.321 (2)
N2—C2	1.351 (2)	N6—H6	0.8600
O2—C6	1.207 (2)	N6—H7	0.8600
N3—C3	1.320 (2)	N5—H4	0.8600
N3—C2	1.355 (2)	N5—H5	0.8600
N4—C1	1.318 (2)	O4—C4	1.232 (2)
N4—H3	0.8600	C4—O3	1.281 (2)
N4—H2	0.8600	O3—H10	1.47 (2)
			
C6—O1—H10	101.7 (12)	C4—C5—H9	107.6
C3—N1—C1	119.22 (15)	H8—C5—H9	107.0
C3—N1—H1	120.4	N5—C2—N2	117.92 (16)
C1—N1—H1	120.4	N5—C2—N3	116.12 (15)
C1—N2—C2	115.93 (15)	N2—C2—N3	125.96 (15)
C3—N3—C2	115.32 (15)	C3—N6—H6	120.0
C1—N4—H3	120.0	C3—N6—H7	120.0
C1—N4—H2	120.0	H6—N6—H7	120.0
H3—N4—H2	120.0	C2—N5—H4	120.0
N4—C1—N2	120.72 (15)	C2—N5—H5	120.0
N4—C1—N1	117.93 (15)	H4—N5—H5	120.0
N2—C1—N1	121.35 (15)	O2—C6—O1	121.35 (17)
N6—C3—N3	121.03 (15)	O2—C6—C5	121.91 (16)
N6—C3—N1	116.77 (15)	O1—C6—C5	116.74 (16)
N3—C3—N1	122.21 (15)	O4—C4—O3	123.80 (18)
C6—C5—C4	118.87 (15)	O4—C4—C5	118.79 (17)
C6—C5—H8	107.6	O3—C4—C5	117.40 (17)
C4—C5—H8	107.6	C4—O3—H10	98.9 (8)
C6—C5—H9	107.6		

### Hydrogen-bond geometry (Å, º)

**Table:**

*D*—H···*A*	*D*—H	H···*A*	*D*···*A*	*D*—H···*A*
N1—H1···O4^i^	0.86	1.82	2.6785 (19)	176
N4—H2···O2^ii^	0.86	2.17	2.8350 (19)	134
N4—H3···N2^ii^	0.86	2.14	2.994 (2)	171
N5—H4···O2	0.86	2.14	2.998 (2)	172
N5—H5···N3^iii^	0.86	2.23	3.091 (2)	178
N6—H6···O1^iii^	0.86	2.15	2.8592 (19)	140
N6—H7···O3^i^	0.86	2.02	2.880 (2)	173
O1—H10···O3	1.00 (2)	1.47 (2)	2.450 (2)	165 (2)

Symmetry codes: (i) *x*−1, *y*, *z*−1; (ii) −*x*, −*y*+1, −*z*+1; (iii) −*x*+2, −*y*+2, −*z*+1.
